# Transient Inhibition of the JNK Pathway Promotes Human Hematopoietic Stem Cell Quiescence and Engraftment

**DOI:** 10.1093/stcltm/szac019

**Published:** 2022-04-15

**Authors:** Huangfan Xie, Zhongjie Sun, Xiong Xiao, Defang Liu, Hailong Qi, Guoxiong Tian, Miao Chen, Ligong Chen, XunCheng Su

**Affiliations:** School of Pharmaceutical Sciences, Key Laboratory of Bioorganic Phosphorus Chemistry and Chemical Biology (Ministry of Education), Tsinghua University, Beijing, People’s Republic of China; State Key Laboratory of Elemento-Organic Chemistry, College of Chemistry, Nankai University, Tianjin, People’s Republic of China; Newish Technology (Beijing) Co. Ltd., Beijing, People’s Republic of China; School of Pharmaceutical Sciences, Key Laboratory of Bioorganic Phosphorus Chemistry and Chemical Biology (Ministry of Education), Tsinghua University, Beijing, People’s Republic of China; Newish Technology (Beijing) Co. Ltd., Beijing, People’s Republic of China; Newish Technology (Beijing) Co. Ltd., Beijing, People’s Republic of China; Newish Technology (Beijing) Co. Ltd., Beijing, People’s Republic of China; Peking Union Medical College Hospital (East), Beijing, People’s Republic of China; School of Pharmaceutical Sciences, Key Laboratory of Bioorganic Phosphorus Chemistry and Chemical Biology (Ministry of Education), Tsinghua University, Beijing, People’s Republic of China; Newish Technology (Beijing) Co. Ltd., Beijing, People’s Republic of China; State Key Laboratory of Elemento-Organic Chemistry, College of Chemistry, Nankai University, Tianjin, People’s Republic of China

**Keywords:** cord blood, hematopoietic stem cell, JNK pathway, quiescence, engraftment

## Abstract

The widespread clinical application of cord blood (CB) for hematopoietic stem cell (HSC) transplantation is limited mainly by the inadequate number of hematopoietic stem and progenitor cells (HSPCs) in single CB units, which results in unsuccessful or delayed engraftment in recipients. The identification of agents to promote CB HSPC engraftment has significant therapeutic value. Here, we found that transient inhibition of the JNK pathway increased the HSC frequency in CB CD34^+^ cells to 13.46-fold. Mechanistic studies showed that inhibition of the JNK pathway upregulated the expression of quiescence-associated and stemness genes in HSCs, preventing HSCs from entering the cell cycle, increasing glucose uptake and accumulating reactive oxygen species (ROS). Importantly, transient inhibition of the JNK pathway during CB CD34^+^ cell collection also enhanced long-term HSC (LT-HSC) recovery and engraftment efficiency. Collectively, these findings suggest that transient inhibition of the JNK pathway could promote a quiescent state in HSCs by preventing cell cycle entry and metabolic activation, thus enhancing the HSC number and engraftment potential. Together, these findings improve the understanding of the regulatory mechanisms governing HSC quiescence and stemness and have the potential to improve HSC collection and transplantation.

## Graphical Abstract

**Figure:**
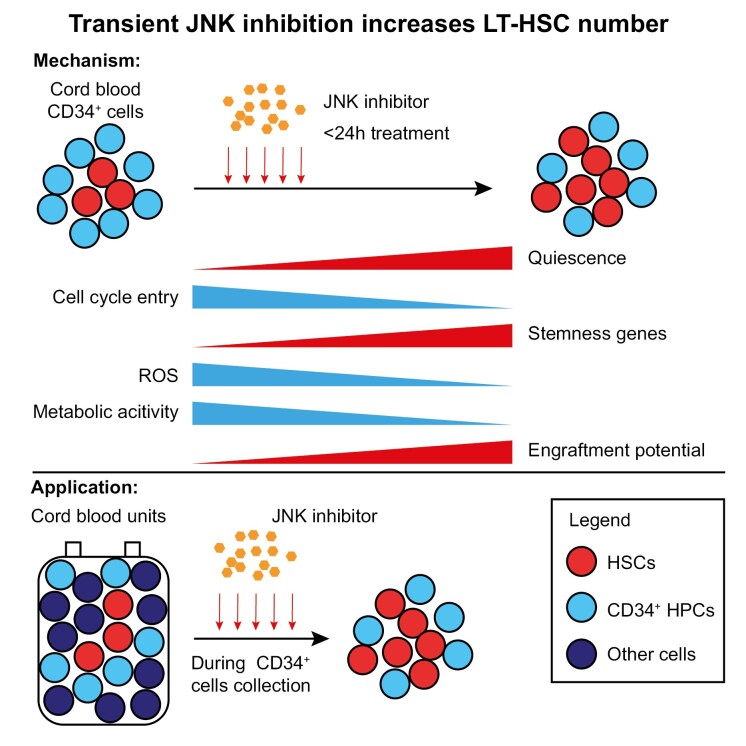

## Introduction

The ability of HSCs to undergo self-renewal and differentiate into almost all blood cell lineages makes HSC transplantation the most commonly used cell therapy to treat malignant and non–malignant hematological disorders.^1^ CB, which has the advantages of a less strict HLA-matching demand, low incidence of graft-versus-host-disease, and low disease relapse rate, is an attractive donor source for HSC transplantation.^2^ Current estimates indicate that there have been more than 40 000 recipients of CB HSC transplantation worldwide^3^; however, the insufficient number of HSCs in single CB units greatly limits the clinical application of CB HSCs.^4^ Multiple efforts have been made to improve the engraftment efficacy of CB HSCs.^4,5^ These approaches primarily include (1) increasing the HSC number by in vitro expansion with cytokines, small molecules^6,7^ or artificial niches^8-10^ and (2) boosting HSC homing with reagents that can enhance HSC homing receptor expression^11-13^ or modify HSC niches in the recipient.^14^ Although several methods^6,8,9^ have significantly improved CB HSC engraftment efficacy, alternative strategies are needed to maximize the functional HSC harvest from single CB units.

The most primitive HSCs with long-term repopulation ability reside in a hypoxic environment and exhibit a quiescent status in their in vivo niche.^15,16^ They barely divide and exhibit rather low metabolic activity with little ROS.^16^ When exposed to cytokines or other ambient stimuli, quiescent HSCs can be activated and enter the cell cycle; however, they always lose their self-renewal ability.^17^ The JNK pathway is heavily involved in the regulation of cell proliferation and metabolic processes.^18^ Therefore, we hypothesized that manipulation of the JNK pathway could improve human HSC engraftment by regulating the transition of the cell cycle and metabolic status.

## Materials and Methods

### Mice and CB

All of the mice used for transplantation were NOD-Prkdcscid Il2rgtm1/Vst (NPG) mice (Stock No.: VS-AM-001) purchased from Beijing Vitalstar Biotechnology and ranged from 8 to 12 weeks of age. CB was obtained from healthy donors upon approval by Peking Union Medical College Hospital. All the animal procedures were performed according to the Animal Protection Guidelines of Tsinghua University, China. All the mouse experiments were approved by the Institutional Animal Care and Use Committee of Tsinghua University. This study was approved by the Institutional Review Board of Peking Union Medical College Hospital (ZS-2483) and conducted according to the approved protocol in compliance with the Declaration of Helsinki.

### Human CB CD34^+^ Cell Isolation and In Vitro Culture

Human CB CD34^+^ cells were isolated with a CD34 MicroBead Kit (Miltenyi Biotec) according to the manufacturer’s instructions and cultured in StemSpan SFEMII (Stem Cell Technologies) supplemented with recombinant human SCF (100 ng/mL, StemImmune LLC), recombinant human FLT3L (100 ng/mL, StemImmune LLC), recombinant human TPO (50 ng/mL, StemImmune LLC), and the indicated molecules. Details are provided in the Supplementary Methods section of the Supplementary material.

### Transplantation and Engraftment Analysis

Cells were transplanted into mouse recipients via the tail vein. Repopulated human cells in NPG mouse peripheral blood (PB), bone marrow (BM), and spleen were monitored by flow cytometry at the indicated time points post-transplantation. Details are provided in the Supplementary Methods section of the Supplementary material.

### Flow Cytometry Analysis

Fresh uncultured cells or cultured cells collected at 24 h were incubated with the indicated antibodies for 30 minutes at 4 °C in PBS containing 0.5% BSA (Sigma–Aldrich, Cat: A1470-100G). Then, the cells were washed with PBS and suspended in 0.2 mL PBS for analysis. Flow cytometry analysis was performed using a FACSCantoTM II (BD). Data were analyzed using FlowJo v10 (BD). Details are provided in the Supplementary Methods section of the Supplementary material.

### Limiting Dilution Analysis

The HSC frequency was quantified by extreme limiting dilution analysis (http://bioinf.wehi.edu.au/software/elda/) with 95% CIs. The engraftment of more than 0.1% of human CD45^+^ cells in the BM was regarded as a positive response.

### Quantification and Statistical Analysis

Statistical analysis was performed with GraphPad Prism software. Data are shown as the mean and SD. Pairwise comparisons between different groups were assessed using an unpaired *t-*test. For all analyses, *P* < .05 was considered statistically significant. The statistical significance and *n* value are reported in the figure legends. All the flow analysis data were processed with FlowJo v10 software. All the figures were prepared with Adobe Illustrator.

## Results and Discussion

### Transient Inhibition of the JNK Pathway was Identified to Increase the HSC Number in CB

**Figure 1.**
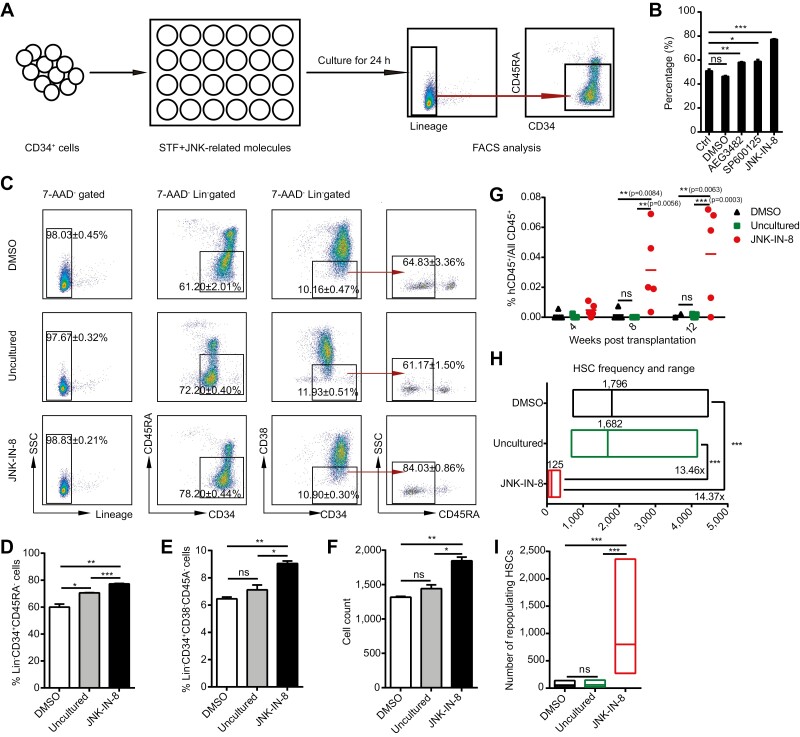
Transient inhibition of the JNK pathway increased the HSC number in CB CD34^+^ cells. (**A**) Schematic of the experimental design. STF represents basic culture medium (StemSpan SFEM II supplemented with 100 ng/mL SCF, 50 ng/mL TPO, and 100 ng/mL Flt3L). Conditions that increased the frequency of Lin^-^CD34^+^CD45RA^−^ cells compared with culturing in basic culture medium were regarded as positive hits. (**B**) Bar plot showing the percentage of Lin^−^CD34^+^CD45RA^−^ cells in CB CD34^+^ cells cultured in medium supplemented with cytokines only (Ctrl), DMSO, AEG3482, SP600125, or JNK-IN-8 for 24 h. (*n* = 3) (**C**) Representative FACS plots showing the expression of the indicated surface markers on DMSO-treated, uncultured, and JNK-IN-8-treated CB CD34^+^ cells. (**D** and **E**) Bar plot showing the frequencies of Lin^−^CD34^+^CD45RA^−^ (**D**) and Lin^−^CD34^+^CD38^−^CD45RA^−^ (**E**) cells in DMSO-treated, uncultured, and JNK-IN-8-treated CB CD34^+^ cells. (**F**) Bar plot showing the Lin^−^CD34^+^CD38^−^CD45RA^−^ cell count in 2 × 10^4^ DMSO-treated, uncultured, and JNK-IN-8-treated CB CD34^+^ cells. (**G**) The frequency of engrafted human CD45^+^ cells in the PB of recipient mice receiving DMSO-treated, uncultured, or JNK-IN-8-treated CB CD34^+^ cells measured at 4-, 8-, and 12-weeks post-transplantation. (**H**) HSC frequencies presented as 1/uncultured CD34^+^ cell equivalent for DMSO-treated, uncultured, and JNK-IN-8-treated CB CD34^+^ cells calculated with ELDA software at 8 weeks post-transplantation. The required CI was 95%. The cutoff for positive engraftment was set as more than 0.01% human CD45^+^ cells in the PB of the recipient (*n* = 5 mice for each group). (**I**) Number of repopulating HSCs per 1 × 10^5^ CD34^+^ cells in the DMSO-treated, uncultured, and JNK-IN-8-treated groups (*n* = 5 mice for each group). See also Supplementary Fig. S1C, S1D; Supplementary Table S1. All data are shown as the mean value ± SD. Statistical significance was assessed using one-way ANOVA if not mentioned. ns, not significant; *P <.05; **P < .01; ***P < .001.

We screened a panel of JNK pathway-related small molecules to increase the CB Lin^−^CD34^+^CD45RA^−^ cell population, which is enriched for HSCs^19^ (Fig. 1A). The results showed that the JNK inhibitors AEG3482, SP600125, and JNK-IN-8, could each significantly increase the frequency of Lin^−^CD34^+^CD45RA^−^ cells after 24 h of incubation compared with control treatment (cytokine-only or DMSO-treated cells) (Fig. 1B). The highest frequency of HSCs was obtained with 15 μM JNK-IN-8 (Supplementary Fig. S1A), which significantly downregulated the expression of the target *c-Jun* (Supplementary Fig. S1B), and there were significant increases in the frequency and number of phenotypic HSC subsets (Lin^−^CD34^+^CD38^−^ and Lin^−^CD34^+^CD38^−^CD45RA^−^ cells) in the JNK-IN-8-treated group compared with the uncultured and DMSO-treated groups (Fig. 1C−1F). Furthermore, after transplantation, the JNK-IN-8-treated group exhibited better engraftment efficiency than the uncultured and DMSO-treated groups (Fig. 1G; Supplementary Table S1). And a limiting dilution assay (LDA) showed that the JNK-IN-8-treated group possessed a higher transplantable HSC frequency, with 13.46-fold and 14.37-fold increases compared with the uncultured and DMSO-treated groups, respectively (Fig. 1H; Supplementary Fig. S1C, S1D); the HSC number was approximately 800 for every 1 × 10^5^ CD34^+^ cells in the JNK-IN-8-treated group, which was higher than the numbers in the uncultured and DMSO-treated groups (60 and 56, respectively) (Fig. 1I). Together, these results suggest that transient inhibition of the JNK pathway significantly enhances phenotypic and transplantable HSC numbers in CB CD34^+^ cells.

**Figure 2.**
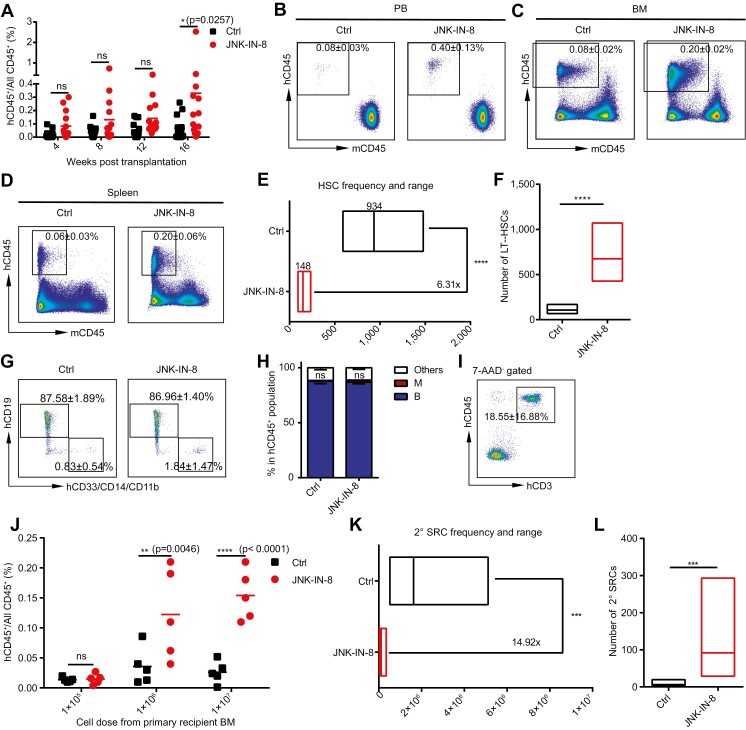
Transient inhibition of the JNK pathway increased the LT-HSC number during CB CD34^+^ cell isolation. (**A**) The frequency of engrafted human CD45^+^ cells in the PB of recipient mice receiving conventionally isolated (Ctrl) or JNK-IN-8-treated CB CD34^+^ cells measured at 4-, 8-, 12-, and 16-weeks post-transplantation. (**B**–**D**) Representative FACS plots showing human CD45^+^ cell engraftment in the PB (**B**), BM (**C**), and spleen (**D**) of recipient mice at 20 weeks after transplantation of conventionally isolated (Ctrl) or JNK-IN-8-treated CB CD34^+^ cells. (**E**) HSC frequencies presented as 1/uncultured CD34^+^ cell equivalent for conventionally isolated (Ctrl) and JNK-IN-8-treated CB CD34^+^ cells calculated with ELDA software at 20 weeks post-transplantation. The required CI was 95%. The cutoff for positive engraftment was set as more than 0.1% human CD45^+^ cells in the BM of the recipient (*n* = 15 mice for each group with 3 independent experiments, ****P < .0001). (**F**) Number of LT-HSCs per 1 × 10^5^ cells in conventionally isolated (Ctrl) and JNK-IN-8-treated CB CD34^+^ cells. See also Supplementary Fig. S2D, S2E and Supplementary Table S2A, S2B.(**G**) Representative FACS plots showing human B (CD19^+^) and myeloid cell (CD33/CD14/CD11b^+^) repopulation in recipient BM (gated in hCD45^+^) at 20 weeks post-transplantation. (**H**) Bar plot showing the lineage distribution of engrafted human CD45^+^ cells in recipient BM. B, CD19^+^ B cells; M, CD33^+^/CD14^+^/CD11b^+^ myeloid cells; *n* = 5. (**I**) Representative FACS plots showing human T-cell (hCD45^+^CD3^+^) repopulation in the recipient thymus at 20 weeks post-transplantation. (**J**) Level of human CD45^+^ cell engraftment in the PB of 2° recipients at the indicated doses of BM cells from primary recipients at 14 weeks post-transplantation. (**K**) 2° SRC frequencies in the BM of the primary recipients receiving conventionally isolated (Ctrl) and JNK-IN-8-treated CB CD34^+^ cells calculated with ELDA software at 14 weeks post-transplantation. The required CI was 95%. The cutoff for positive engraftment was set as more than 0.01% human CD45^+^ cells in the PB of the recipient (*n* = 5 mice for each group, ***P < .001). (**L**) Number of 2° SRC per 1 × 10^7^ cells in conventionally isolated (Ctrl) and JNK-IN-8-treated CB CD34^+^ cells. See also Supplementary Fig. S2F, S2G and Supplementary Table S2C. All data are shown as the mean value ± SD. Statistical significance was assessed using one-way ANOVA if not mentioned. ns, not significant; *P < .05; **P < .01; ***P < .001; ****P < .0001.

We next investigated whether transient inhibition of the JNK pathway during CD34^+^ cell isolation can increase LT-HSC harvest from single CB units by the LDA, in which 2000/500/200 conventionally isolated CD34^+^ cells (control group) and the same doses of JNK-IN-8-treated CD34^+^ cells were injected into separate immunodeficient NPG mice. We found that the JNK-IN-8-treated group exhibited better engraftment efficiency than the control group (Fig. 2A; Supplementary Table S2A, S2B), with human CD45^+^ cell engraftment being higher in the PB (Fig. 2B; Supplementary Fig. S2A), BM (Fig. 2C; Supplementary Fig. S2B) and spleen (Fig. 2D; Supplementary Fig. S2C) of the recipients at 20 weeks post-transplantation. The calculated HSC frequency was increased in the JNK-IN-8-treated group compared with the control group (1/148 vs. 1/934; Fig. 2E; Supplementary Fig. S2D, S2E); that is, approximately 676 LT-HSCs were harvested in the JNK-IN-8-treated group for every 1 × 10^5^ CD34^+^ cells, while the LT-HSC number of the control group was only approximately 107 (Fig. 2F). JNK-IN-8 treatment did not alter the B/myeloid cell ratio of engrafted human cells (Fig. 2G, 2H), and CD3^+^ T cells could be detected in the thymus (Fig. 2I). Moreover, after secondary transplantation, the BM cells from the JNK-IN-8-treated group provided better engraftment than the control group (Fig. 2J). The LDA showed that the 2° SCID-repopulating cells (SRCs) frequency of the JNK-IN-8-treated group was 14.92-fold higher than that of the control group (approximately 92 in the JNK-IN-8-treated group versus 6 in the control group for every 1 × 10^7^ primary recipient BM cells) (Fig. 2K, 2L; Supplementary Fig. S2F, S2G; Supplementary Table S2C). Collectively, these results suggest that JNK-IN-8 treatment can enhance LT-HSC harvest from CB units without altering the HSC multilineage repopulation pattern.

### Transient Inhibition of the JNK Pathway Promoted Quiescent HSC-Specific Gene Expression Profiles

**Figure 3.**
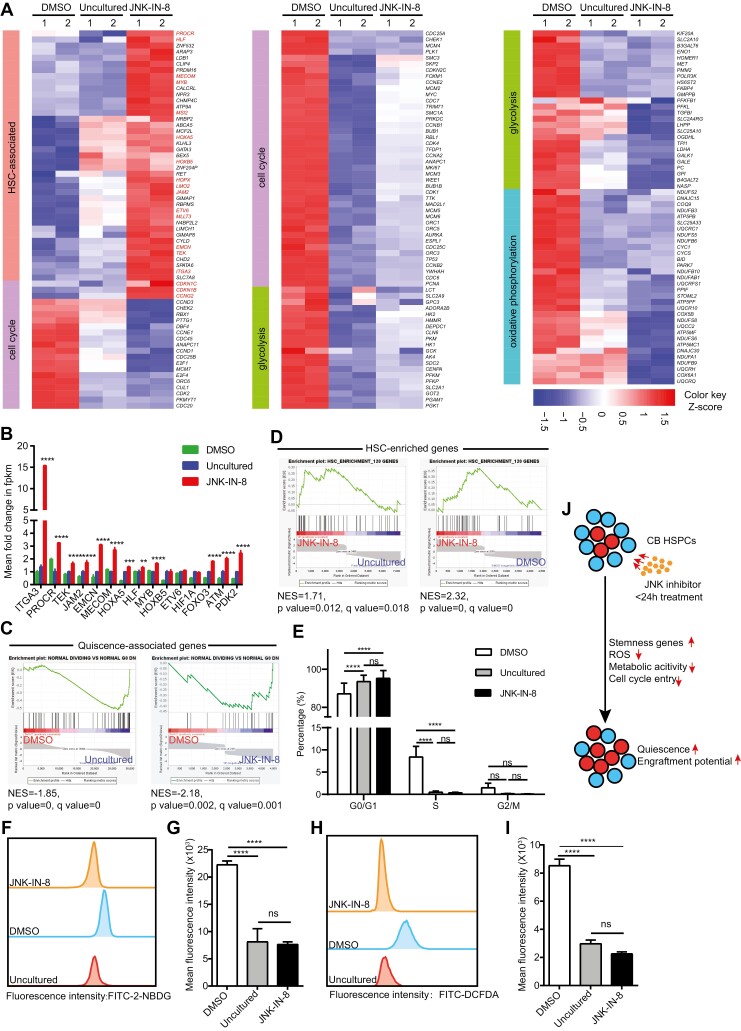
Transient inhibition of the JNK pathway promoted HSC quiescence, preventing HSCs from undergoing cell cycle entry and metabolic activation. (**A**) Heatmap showing gene expression in DMSO-treated, uncultured, and JNK-IN-8-treated CB CD34^+^ cells. (**B**) Expression by RNA-seq of the indicated genes in DMSO-treated, uncultured, and JNK-IN-8-treated CB CD34^+^ cells. The fpkm value for uncultured cells is normalized to 1.0; *n* = 2. (**C** and **D**) GSEA plots showing enrichment of quiescent-HSC-enriched (**C**) and 120 HSC-associated (**D**) gene sets in the indicated groups. DMSO, DMSO-treated CB CD34^+^ cells; uncultured, uncultured CB CD34^+^ cells; JNK-IN-8, JNK-IN-8-treated CB CD34^+^ cells. Each group contained 2 replicates. (**E**) Bar plot showing the cell cycle status of DMSO-treated, uncultured, and JNK-IN-8-treated CB CD34^+^ cells (*n* = 3). (**F** and **G**) Representative FACS plots (**F**) and bar plot (**G**) showing glucose uptake activity (indicated by the fluorescence intensity of 2-NBDG) of DMSO-treated, uncultured, and JNK-IN-8-treated CB CD34^+^ cells (*n* = 3). (**H** and **I**) Representative FACS plots (**H**) and bar plot (**I**) showing ROS levels (indicated by the fluorescence intensity of DCFDA) of DMSO-treated, uncultured, and JNK-IN-8-treated CB CD34^+^ cells (*n* = 3). (**J**) Schematic of the proposed model demonstrating how transient JNK inhibition regulates CB HSPC engraftment through its roles in quiescence and stemness. All data are shown as the mean value ± SD. Statistical significance was assessed using one-way ANOVA if not mentioned. ns, not significant; **P <.01; ***P < .001; ****P < .0001.

To investigate the mechanism by w JNK-IN-8 treatment increased HSC numbers, we compared the transcription profiles of DMSO-treated, uncultured, and JNK-IN-8-treated CB CD34^+^ cells generated by RNA sequencing (RNA-seq). Above all, JNK-IN-8-treated cells exhibited upregulation of quiescent cell-enriched gene set expression (Fig. 3A–3C). Specifically, compared with uncultured and DMSO-treated cells, JNK-IN-8-treated cells upregulated HSC-associated gene expression (Fig. 3A, 3B): the surface markers enriched on primitive HSCs such as *ITGA3*,^20^*PROCR*,^21^*TEK*,^22^*JAM2*,^23^ and *EMCN*^24^ were highly expressed after JNK-IN-8 treatment, and the expression of key genes involved in HSC stemness and self-renewal abilities,^17^ including *MECOM*, *HOXA5*, *HLF*, *MYB*, *HOXB5*, *ETV6*, *MLLT3*, *MSI2, and HOPX*, was also upregulated in the JNK-IN-8-treated group. Moreover, gene set enrichment analysis (GSEA) showed that most of the previously reported 120 HSC-specific genes^21^ were upregulated in the JNK-IN-8-treated group (Fig. 3D).

We next explored cell cycle- and metabolism-related gene expression. We found that DMSO-treated cells had highly upregulated cell cycle entry genes and active genes involved in glycolysis and oxidative phosphorylation compared with uncultured cells, while the expression levels of these genes were maintained or reduced in JNK-IN-8-treated cells (Fig. 3A, 3B). Notably, JNK-IN-8 treatment upregulated cell cycle inhibitor (*CDKN1B*, *CDKN1C,* and *CCNG2*) expression (Fig. 3A; Supplementary Fig. S3E). Likewise, GSEA revealed that the expression of E2F and MYC targets was upregulated in the DMSO-treated group but downregulated in the JNK-IN-8-treated group (Supplementary Fig. S3A, S3B). Similar results were also observed for glycolysis and oxidative phosphorylation gene sets (Supplementary Fig. S3C–S3E).

### Transient Inhibition of the JNK Pathway Prevented HSCs From Undergoing Cell Cycle Entry and Metabolic Activation

We further conducted cell cycle status analysis, glucose uptake activity measurement, and ROS level detection with DMSO-treated, uncultured, and JNK-IN-8-treated CB CD34^+^ cells. The DMSO-treated group showed a much higher percentage of cells in the S phase, while the JNK-IN-8-treated and uncultured groups consisted mostly of G0/G1 cells (Fig. 3E). DMSO-treated cells showed the highest glucose uptake activity and intracellular ROS level, while JNK-IN-8-treated and uncultured cells maintained similarly low levels (Fig. 3F–3I). These results, together with the gene expression profile analysis (Fig. 3A–3D; Supplementary Fig. S3), indicate that JNK-IN-8 treatment promotes the quiescence status of HSCs by preventing HSCs from undergoing cell cycle entry and metabolic activation (Fig. 3J).

We have previously reported a supportive role for JNK inhibition in HSC self-renewal in vitro; however, the engraftment process was slow, and the detailed mechanism was largely unclear.^25^ Here, by increasing the concentration of JNK inhibitors (15 μM) and shortening the incubation period (24 h), we obtained a greater increase in the HSC number (13.46-fold). We found, for the first time, a novel role for JNK pathway inhibition in the promotion of HSC quiescence and stemness.

In our study, transient JNK inhibition modestly increased the phenotypic HSC (Lin^−^CD34^+^CD38^−^CD45RA^−^) number in CB CD34^+^ cells, but the transplantable HSC number was dramatically increased. There is no immunophenotype that can identify functional HSCs with 100% purity; therefore, engraftment efficiency is the gold standard to evaluate functional HSCs. In our context, CD34^+^ cells were a remarkably heterogeneous population, making the exact identity of the target cells affected by JNK inhibition unclear. Considering that the majority of cells were in the G0/G1 phase, we postulated that this notable increase in functional HSCs probably occurred through rapid enhancement of latent HSC activity in CB HSPCs rather than expansion. Transient JNK inhibition markedly enhanced the expression of key HSC-specific stemness genes, including the surface markers *TEK*, *PROCR*, and *ITGA3* and the transcription factors *MECOM*, *HOXA5*, *HLF*, *MYB*, *HOXB5*, *ETV6*, *MLLT3*, *MSI2*, and *HOPX*; HSCs exhibited low metabolic activity and reduced ROS levels under JNK inhibition. Each of the characteristics above has been reported in HSCs showing the greatest engraftment potential. Therefore, we hypothesized that transient JNK inhibition enhances CB HSC engraftment ability by recruiting cells in the CD34^+^ compartment that would not otherwise engraft via upregulation of stemness genes and promotion of quiescence.

## Conclusion

In summary, we observed a high increase in the HSC number (13.46-fold) in CB CD34^+^ cells after incubation with JNK-IN-8 (15 µM) for 24 h. We also found a novel role for JNK pathway inhibition in the promotion of HSC quiescence and stemness. More interestingly, we found that supplementation with JNK inhibitors during CD34^+^ cell collection could significantly increase the LT-HSC number in CB units, which could help maximize functional HSC harvest from CB in clinical settings.

Our study extends the understanding of HSC quiescence and stemness regulation. Further investigation of whether transient inhibition of the JNK pathway can complement previous endeavors to maximize functional HSC harvest from single CB units using a combinational approach is needed.

## Supplementary Material

szac019_suppl_Supplementary_Material

## Data Availability

All software used in this study is listed in the methods sections and available online. The RNA-seq data have been deposited at GEO (accession number GSE165501). For original data, please contact ligongchen@tsinghua.mail.com.
