# Correction to: Transient Inhibition of the JNK Pathway Promotes Human Hematopoietic Stem Cell Quiescence and Engraftment

**DOI:** 10.1093/stcltm/szad001

**Published:** 2023-01-30

This is a correction to: Huangfan Xie, Zhongjie Sun, Xiong Xiao, Defang Liu, Hailong Qi, Guoxiong Tian, Miao Chen, Ligong Chen, XunCheng Su, Transient Inhibition of the JNK Pathway Promotes Human Hematopoietic Stem Cell Quiescence and Engraftment, *Stem Cells Translational Medicine*, Volume 11, Issue 6, June 2022, Pages 597–603, https://doi.org/10.1093/stcltm/szac019

In the originally published version of this article, the institutional affiliations labeled 1 and 2 were presented in the wrong order. These should have been presented as follows:

1. State Key Laboratory of Elemento-organic Chemistry, College of Chemistry, Nankai University, Tianjin 300071, China

2. School of Pharmaceutical Sciences, Key Laboratory of Bioorganic Phosphorus Chemistry and Chemical Biology (Ministry of Education), Tsinghua University, Beijing 100084, China

This error has been corrected.

