# Supplementary material for: Transient Inhibition of the JNK Pathway Promotes Human Hematopoietic Stem Cell Quiescence and Engraftment
Source: Stem Cells Transl Med. 2022 Apr 15;11(6):597–603. doi: 10.1093/stcltm/szac019 (PMC9216500; doi:10.1093/stcltm/szac019)
Supplement: szac019_suppl_Supplementary_Material [file szac019_suppl_supplementary_material.pdf]

Supplemental Data

Supplemental Methods

Human cord blood $CD34^+$ cell isolation

Human cord blood (CB) $CD34^+$ cells were isolated with density gradient centrifugation and a CD34 MicroBead Kit (Miltenyi Biotec, Cat: 130-046-703) according to the manufacturer’s instructions. Briefly, each CB unit was diluted with an equal volume of sterile phosphate-buffered saline (PBS; Kai na bo China, Cat: C0160) in T75 flasks and then added gently to Ficoll-Paque™ PLUS (GE Healthcare Biosciences, Cat: 17-1440-03) in a 50-ml tube, ending with 35 ml diluted CB and 15 ml Ficoll-Paque™ PLUS in each tube. The tubes were centrifuged at 500 g for 30 min according to the manufacturer’s protocol. Mononuclear cells were collected from the liquid interface and washed three times with PBS through centrifugation at 500 g for 5 min. Then, the mononuclear cells were stained with equivalent $CD34^+$ MicroBeads and FcR Blocking Reagent (Miltenyi Biotec, Cat: 130-046-702) at 4 °C for 30 min. The incubated cells were washed twice with PBS. Then, the cell suspension was loaded onto a MACS® Column, which was placed in the magnetic field of a MACS® MultiStand (Miltenyi Biotec, Cat: 130-042-303). After removing the column from the magnetic field, the magnetically retained $CD34^+$ cells were eluted into a new tube with PBS as the positively selected cell fraction. When $CD34^+$ cells were isolated with JNK inhibitors, all the reagents used during the isolation process were supplemented with JNK-IN-8 at a concentration of 5 μM.

In vitro culture

Human CB CD34+ cells were cultured in StemSpan SFEMII (Stem Cell Technologies, Cat: 09655) supplemented with recombinant human SCF (100 ng/ml, StemImmune LLC, Cat: HHM-SF-0100), recombinant human FLT3L (100 ng/ml, StemImmune LLC, Cat: HHM-FT-0100) and recombinant human TPO (50 ng/ml, StemImmune LLC, Cat: HHM-TP-0100). CB CD34+ cells were cultured in suspension in ultralow-attachment 24-well or 6-well plates (Corning) at 37 °C and 5% CO_2 in a culture incubator. JNK-IN-8 (15 μM, Selleck, Cat: S4901) dissolved in DMSO (Sigma–Aldrich, Cat: D2650) was added to the culture medium, and 0.01% DMSO was added as the control condition. CB CD34+ cells were collected after 24 h of culture for transplantation, flow cytometry analysis and functional assays. Other compounds including SP600125 (5 μM, Selleck, Cat: S1460), Tanzisertib (2 μM, Selleck inhibitor, Cat: S8490), AEG3482 (10 μM, Tocris, Cat: 2651), TCS JNK 6o (1 μM, Tocris, Cat: 3222), the JNK inhibitor IX (0.1 μM, Selleck inhibitor, Cat: S7508) and BI-78D3 (2 μM, Selleck inhibitor, Cat: S8201) were used.

To identify the optimal concentration of JNK-IN-8, CD34+ cells were cultured with JNK-IN-8 at the concentrations of 2 μM, 5 μM, 10 μM, 15 μM, 20 μM, 25 μM and 50 μM for 24 h.

Flow cytometry analysis

The following reagents and antibodies were used for phenotypic HSPC analysis: 7-AAD (Sino Biological, Cat: APK10448-P), FITC-conjugated anti-human CD34

(BioLegend, Cat: 343604), PE-conjugated anti-human CD38 (BioLegend, Cat: 356604), and APC-Cy7-conjugated anti-human CD45RA (BioLegend, Cat: 304128). Lineage antibody cocktail was the mixture of the following antibodies: PE-Cy7-conjugated anti-human CD3 (BioLegend, Cat: 100219), PE-Cy7-conjugated anti-human CD14 (BioLegend, Cat: 367111), PE-Cy7-conjugated anti-human CD16 (BioLegend, Cat: 302015), PE-Cy7-conjugated anti-human CD19 (BioLegend, Cat: 363011), PE-Cy7-conjugated anti-human CD20 (BioLegend, Cat: 302311), PE-Cy7-conjugated anti-human CD56 (BioLegend, Cat: 362509).

For analysis of engrafted human hematopoietic lineages, PB cells were collected at the indicated times; the BM and spleen were isolated at 20 weeks post-transplantation. Cells were stained with the following reagents and antibodies: 7-AAD, PE-Cy7-conjugated anti-human CD45 (BioLegend, Cat: 304016), FITC-conjugated anti-mouse CD45 (BioLegend, Cat: 103108), PE-conjugated anti-human CD19 (BioLegend, Cat: 302208), APC-conjugated anti-human CD11b (BioLegend, Cat: 301310), APC-conjugated anti-human CD33 (BioLegend, Cat: 366606) and APC-conjugated anti-human CD14 (BioLegend, Cat: 301808). For detection of T cells in the thymus, cells were stained with the following reagents and antibodies: 7-AAD, PE-Cy7-conjugated anti-human CD45, FITC-conjugated anti-mouse CD45, and PE-Cy5-conjugated anti-human CD3 (BioLegend, Cat: 300309).

Transplantation and monitoring of CB CD34$^+$ cells in NOD-Prkdcscid Il2rgtm1/Vst (NPG) mice

Mice were sublethally irradiated 3-6 h before transplantation. Upon transplantation, the mice were immobilized with a special animal fixator. Fresh uncultured CB CD34$^+$ cells or their progeny present in 24-h cultures were transplanted into the mouse recipients via the tail vein with a 29-G insulin syringe (BD, Cat: 320310). For the limiting dilution assay (LDA), collected CD34$^+$ cells prepared with JNK-IN-8 treatment were injected into immunodeficient mice at a dose of 2,000/500/200 or 50 cells per recipient; CD34$^+$ cells prepared by the conventional isolation process without JNK-IN-8 and administered at the same dose were used as the control group. Repopulated human cells in the NPG mouse peripheral blood (PB), bone marrow (BM) and spleen were monitored by flow cytometry at the indicated time points post-transplantation. PB cells were collected from the tail vein of the mouse recipients. At 20 weeks post-transplantation, BM cells were collected from the recipient mice by flushing the two femurs with PBS; recipient spleens and thymuses were ground and washed with PBS into collection tubes and subjected to red blood cell (RBC) lysis using RBC lysis buffer (BioLegend, Cat: 420301) according to the manufacturer's protocol. All the BM, spleen and thymus cells were then filtered through separate 40-μm cell strainers to obtain the corresponding single-cell suspensions. Then, the proportion of repopulated human cells in the recipient PB, BM, spleen or thymus was analyzed according to the flow cytometry analysis protocol.

Secondary Transplantation

At 20 weeks post-transplantation, the primary NPG mouse recipients were sacrificed

and BM were flushed out. The BM engrafted with human $CD45^+$ cells (≥0.1% human chimerism) were resuspended in PBS and transplanted into the secondary NPG mouse recipients via the tail vein. For LDA, primary recipient BM cells were injected at a dose of 1 x 10^7, 1 x 10^6 or 1 x 10^5 cells per recipient. The proportion of repopulated human cells in the recipient PB were assessed with flow cytometry after further 14 weeks.

RNA isolation, cDNA synthesis and real-time qPCR

RNA isolation was performed using an RNeasy Plus Mini Kit (Qiagen, Cat: 74134) according to the manufacturer's protocol. cDNA was prepared using a HiScript® 1st Strand cDNA Synthesis Kit (Vazyme, Cat: R312-02). qPCR was performed with SYBR Green I Master Mix (CLJC-Bio, Cat: Q311-02) on a 7500 Fast Real Time PCR system (Thermo Fisher). The PCR protocol was as follows: first, 94 °C for 2 min to activate the polymerase, followed by 40 cycles at 94 °C for 15 s (for denaturation) and 60 °C for 30 s (for annealing and extension). Values for mRNA expression were normalized to the expression of *β-actin*. The primer sets used to detect single genes are listed in Supplemental Table 3.

RNA-sequencing (RNA-seq) analysis

Total RNA from 1 x 10^6 CB $CD34^+$ cells was extracted using an RNeasy Plus Mini Kit (Qiagen, Cat: 74134). Sequencing libraries were generated using a NEBNext® UltraTM RNA Library Prep Kit for Illumina® (NEB, USA) following the manufacturer's

recommendations, and index codes were added to attribute sequences to each sample. Clustering of the index-coded samples was performed on a cBot Cluster Generation System using a HiSeq 4000 PE Cluster Kit (Illumina) according to the manufacturer's instructions. After cluster generation, the library preparations were sequenced on an Illumina HiSeq 4000 platform, and 150-bp paired-end reads were generated. All the sequencing was performed at Novogene Company in China.

For abundance estimations (FPKMs), aligned read files were further processed with an HT-seq software assay on the hg38 annotation. The selection of differentially expressed genes was based on an adjusted P value <0.05 (significant across two replicates) with De-seq software. Heatmaps were generated using hierarchical clustering with a color scale normalized by log10(FPKM+1) for each gene.

For gene set enrichment analysis (GSEA), differentially expressed genes for which the absolute value of the fold change was more than 4-fold with significance (P value <0.05) between two sample groups were selected and subjected to analysis with GSEA software (version 4.1.8).

Cell cycle analysis

CB $CD34^+$ cells were cultured in culture plates at 5 x 10^4 cells per well with JNK-IN-8 (15 µM) or DMSO (0.01%) for 24 h. Then, these cells or fresh uncultured CB $CD34^+$ cells were collected into 15-ml tubes and centrifuged at 500 g for 5 min. The cell pellet was resuspended by adding 4.5 ml prechilled 70% cold ethanol (-20 °C) in a dropwise manner and gently pipetting. The fixed cells were incubated at 4 °C overnight and then

centrifuged at 600 g for 5 min. Upon ethanol removal, the cells were rinsed with 5 ml PBS by centrifuging at 500 g for 5 min. The supernatant was discarded, and the cells were resuspended in propidium iodide (PI) staining buffer consisting of PI (100 µg/ml, Solarbio, Cat: C0080), RNase A (100 µg/ml, Solarbio, Cat: R1030) and Triton™ X-100 (1%, Sigma–Aldrich, Cat: V900602). After incubation at 37 °C for 30 min, the cells were washed with PBS, and the fluorescence was analyzed by flow cytometry.

Glucose uptake assays

For measurement of glucose uptake, CB CD34$^+$ cells were collected and resuspended in RPMI 1640 medium supplement with 2% fetal bovine serum (FBS) and then incubated with 2-(*N*-(7-nitrobenz-2-oxa-1,3-diazol-4-yl)amino (2-NBDG; 50 µM, Invitrogen, Cat: N13195) at 37 °C with 5% CO_2 for 4 h. After the incubation, the cells were collected, washed once with prechilled PBS, resuspended in analysis buffer and then analyzed by flow cytometry.

Measurement of cellular reactive oxygen species (ROS)

To measure intracellular ROS levels, CB CD34$^+$ cells were collected and incubated at 37 °C in the dark for 30 min with 5 µM 2’,7’-dichlorodihydrofluorescein diacetate (DCFDA; Invitrogen, Cat: C2938). The fluorescence of the cells was then analyzed by flow cytometry.

Supplemental Figure Legends

Supplemental Figure 1. Transient inhibition of the JNK pathway increased the HSC number in CB CD34$^+$ cells. (A) Fitting curve showing the percentage of Lin$^-$ CD34$^+$CD45RA$^-$ cells in CB CD34$^+$ cells cultured with different concentrations of JNK-IN-8 for 24 h (n=3). (B) Real-time qPCR results for *cJun* expression in DMSO-treated and JNK-IN-8-treated CB CD34$^+$ cells. The expression value was normalized to that of *β-actin* (n=6). Statistical significance was assessed using an unpaired *t* test, where *p<0.05. (C and D) Table summarizing the human CD45$^+$ cell engraftment (C) and LDA plot showing the HSC frequency (D) of DMSO-treated, uncultured and JNK-IN-8-treated CB CD34$^+$ cells calculated with ELDA software at 8 weeks post-transplantation. The required confidence interval was 95% (n=5 mice for each group).

Supplemental Figure 2. Transient inhibition of the JNK pathway during CB CD34$^+$ cell isolation increased the LT-HSC harvest. (A-C) The frequency of engrafted human CD45$^+$ cells in the PB (A), BM (B) and spleen (C) of recipient mice receiving conventionally isolated (Ctrl) or JNK-IN-8-treated CB CD34$^+$ cells measured at 20 weeks post-transplantation. Statistical significance was assessed using an unpaired *t* test. (D and E) Table summarizing the human CD45$^+$ cell engraftment (D) and LDA plot showing the 1° SRC frequency (E) of conventionally isolated (Ctrl) and JNK-IN-8-treated CB CD34$^+$ cells calculated with ELDA software at 20 weeks post-transplantation. The required confidence interval was 95% (n=15 mice for each group with 3 independent experiments). (F and G) Table summarizing the human CD45$^+$ cell

engraftment in 2° recipients (F) and LDA plot showing the 2° SRC frequency (G) of conventionally isolated (Ctrl) and JNK-IN-8-treated CB $CD34^+$ cells calculated with ELDA software at 14 weeks post-transplantation. The required confidence interval was 95% (n=5 mice for each group).

Supplemental Figure 3. Transient inhibition of the JNK pathway promoted quiescent HSC-specific gene expression profiles. (A-D) GSEA plots showing the enrichment of E2F target (A), MYC target (B), oxidative phosphorylation-related (C) and glycolysis (D)-related gene sets in the indicated groups. DMSO, DMSO-treated CB $CD34^+$ cells; Uncultured, uncultured CB $CD34^+$ cells; JNK-IN-8, JNK-IN-8-treated CB $CD34^+$ cells. Each group contained two replicates. (E) qPCR results for cell cycle- (*CDKN1B*, *CDKN1C* and *CCNG2*) and HSC quiescence-related (*HIF1A*, *FOXO3*, *ATM*, and *PDK2*) genes in DMSO-treated, uncultured and JNK-IN-8-treated CB $CD34^+$ cells. (n=3) All data are shown as the mean value ± SD. Statistical significance was assessed using one-way ANOVA if not mentioned. **p<0.01; ***p<0.001; ****p < 0.0001.

Supplemental Tables

Supplemental Table 1. Summary of human CD45+ cell engraftment in mice receiving DMSO-treated, uncultured or JNK-IN-8-treated CB CD34+ cells.

For limiting dilution analysis of NPG engraftment, human CD45+ cell engraftment in recipient PB were measured at 4-, 8- and 12-weeks post-transplantation.

CD34+ cell dose	Group	% human CD45+ reconstitution in PB		
		4 week	8 week	12 week
5000	DMSO	0.01600	0.03100	0.21000
		0.02400	0.08700	
		0.16000	0.12000	
		0.06200	0.04900	
		0.07200		
	Uncultured	0.02700	0.09300	0.06700
		0.01800	0.13000	0.28000
		0.11000	0.15000	0.08900
		0.02500	0.16000	0.67000
		0.02900	0.00388	0.00456
	JNK-IN-8	0.00841	0.01900	0.03300
		0.02000	0.08200	0.03200
		0.03900	0.01400	
		0.09000	0.06500	
		0.02700	0.09700	
1000	DMSO	0.00603	0.00211	0.01100
		0.00283	0.03600	0.07800
		0.00557	0.00000	0.02800
		0.00323	0.00217	0.03800
		0.03100	0.07600	0.14000
	Uncultured	0.00000	0.06500	0.11000
		0.03800	0.01000	0.09600
		0.00553	0.19000	0.08800
		0.00000	0.02600	0.02200
	JNK-IN-8	0.00000	0.09000	
		0.01400		
		0.04000		
		0.00000		
		0.03000		
200	DMSO	0.00000	0.00000	0.00000
		0.00000	0.00000	0.00215
		0.00000	0.00000	
		0.00578	0.00000	
		0.00000	0.00745	
	Uncultured	0.00000	0.00000	0.00181
		0.00234	0.00000	0.00215
		0.00000	0.00000	0.00000
		0.00000	0.00000	0.00000
		0.00000	0.00000	0.00000
	JNK-IN-8	0.00302	0.01900	0.01300
		0.00253	0.00353	0.00000
		0.00744	0.02000	0.06800
		0.01100	0.06900	0.05800
		0.00000	0.04600	0.07200

Supplemental Table 2. Summary of human CD45^{+} cell engraftment in mice receiving conventionally isolated (Ctrl) and JNK-IN-8-treated CB CD34^{+} cells. For limiting dilution analysis of NPG engraftment, human CD45^{+} cell engraftment in 1°recipient PB (A) were measured at 4-, 8- 12-, 16- and 20-weeks post-transplantation, BM (B) were measured at 20 weeks post-transplantation; 2°recipient PB (C) were measured at 14 weeks post-transplantation.

(A)

CD34+ cell dose	% human CD45+ reconstitution in PB of 1° recipient									
	4 week		8 week		12 week		16 week		20 week	
	Ctrl	JNK-IN-8	Ctrl	JNK-IN-8	Ctrl	JNK-IN-8	Ctrl	JNK-IN-8	Ctrl	JNK-IN-8
2000	0.22000	1.06000	0.86000	1.54000	6.72000	3.15000	1.70000	5.50000	2.98000	3.61000
	0.15000	0.25000	0.74000	1.00000	3.36000	1.21000	1.57000	2.74000	1.75000	1.37000
	0.09000	0.21000	0.62000	0.60000	0.75000	0.94000	0.30000	0.41000	0.74000	1.24000
	0.08700	0.15000	0.60000	0.57000	0.58000	0.43000	0.30000	0.34000	0.43000	0.48000
	0.06400	0.10000	0.25000	0.55000	0.27000	0.37000	0.23000	0.28000	0.28000	0.47000
	0.05500	0.09400	0.22000	0.50000	0.22000	0.25000	0.19000	0.23000	0.17000	0.46000
	0.05100	0.08000	0.18000	0.37000	0.20000	0.24000	0.17000	0.23000	0.14000	0.29000
	0.04000	0.06900	0.11000	0.32000	0.19000	0.22000	0.16000	0.21000	0.11000	0.12000
	0.03700	0.06900	0.11000	0.15000	0.13000	0.21000	0.12000	0.19000	0.02200	
	0.03200	0.05700	0.11000	0.15000	0.09900	0.16000	0.09100	0.18000	0.00000	
	0.02100	0.03900	0.08800	0.14000	0.08500	0.11000	0.06900	0.15000		
	0.01900	0.02700	0.08800	0.05700	0.05600	0.06600	0.05300	0.14000		
	0.00000	0.00000	0.04400	0.04000	0.05100	0.02500	0.05100	0.08800		
	0.00000		0.00000		0.04300		0.02900			
	0.00000		0.00000		0.00417		0.00238			
500	0.09700	0.30000	0.16000	0.73000	0.16000	0.43000	0.26000	2.53000	0.23000	1.43000
	0.07400	0.26000	0.07700	0.35000	0.15000	0.32000	0.21000	0.46000	0.14000	0.47000
	0.03000	0.20000	0.06500	0.26000	0.15000	0.24000	0.17000	0.38000	0.10000	0.41000
	0.02900	0.14000	0.04900	0.19000	0.09200	0.22000	0.07500	0.31000	0.10000	0.25000
	0.02000	0.08100	0.04100	0.09600	0.06300	0.12000	0.07400	0.29000	0.09000	0.24000
	0.00968	0.05100	0.03700	0.06800	0.03700	0.12000	0.05700	0.18000	0.04600	0.24000
	0.00967	0.03100	0.03200	0.06800	0.03100	0.11000	0.04900	0.17000	0.00101	0.23000
	0.00961	0.03100	0.02000	0.02900	0.01700	0.09600	0.03800	0.09600	0.00000	0.19000
	0.00000	0.02100	0.00995	0.01900	0.01000	0.09200	0.00809	0.08000	0.00000	0.13000
	0.00000	0.02100	0.00564	0.00878	0.00259	0.07400	0.00413	0.05500		
	0.00000	0.01000	0.00228	0.00799	0.00000	0.06300	0.00205	0.03000		
	0.00000	0.01000	0.00000	0.00589	0.00000	0.06300	0.00000	0.02700		
	0.00000	0.00990	0.00000	0.00559	0.00000	0.05900	0.00000	0.01600		
	0.00000	0.00000	0.00000	0.00392	0.00000	0.03000	0.00000	0.00457		
200	0.07300	0.05800	0.23000	0.27000	0.35000	0.27000	0.29000	0.33000	0.41000	0.40000
	0.06300	0.05500	0.15000	0.22000	0.17000	0.23000	0.23000	0.32000	0.39000	0.32000
	0.04000	0.04800	0.13000	0.18000	0.14000	0.18000	0.14000	0.24000	0.12000	0.29000
	0.03500	0.04300	0.09400	0.09200	0.08600	0.18000	0.08500	0.17000	0.03500	0.29000
	0.03000	0.03900	0.07100	0.07200	0.08400	0.12000	0.05400	0.15000	0.00745	0.25000
	0.01600	0.03200	0.06600	0.06700	0.06200	0.07500	0.04700	0.12000	0.00731	0.21000
	0.01400	0.03000	0.04600	0.06400	0.05200	0.06900	0.04100	0.12000	0.00602	0.20000
	0.01100	0.02000	0.04500	0.05900	0.04800	0.04600	0.03800	0.10000	0.00000	0.19000
	0.00980	0.01900	0.02100	0.02800	0.04700	0.04400	0.03000	0.09300	0.00000	0.17000
	0.00000	0.01500	0.01500	0.01700	0.01300	0.03300	0.02600	0.07100	0.00000	0.01100
	0.00000	0.01200	0.00000	0.00964	0.00318	0.02400	0.01400	0.06800		
	0.00000	0.00958	0.00000	0.00955	0.00209	0.02200	0.01000	0.06000		
	0.00000	0.00806	0.00000	0.00612	0.00000	0.01600	0.00264	0.03400		
	0.00000	0.00000	0.00000	0.00000	0.00000	0.01300	0.00204	0.00838		
	0.00000	0.00000	0.00000	0.00000	0.00000	0.00000	0.00000	0.00236		
50	0.00000	0.01000	0.00904	0.02100	0.00606	0.32000	0.00485	1.47000	0.00178	0.55000
	0.00000	0.00000	0.00000	0.00000	0.00428	0.20000	0.00204	0.37000	0.00105	0.06400
	0.00000	0.00000	0.00000	0.00000	0.00231	0.02500	0.00000	0.04200	0.00000	0.05200
	0.00000	0.00000	0.00000	0.00000	0.00000	0.00416	0.00000	0.01600	0.00000	0.00196
	0.00000	0.00000	0.00000	0.00000	0.00000	0.00000	0.00000	0.00472	0.00000	0.00000
	0.00000	0.00000	0.00000	0.00000	0.00000	0.00000	0.00000	0.00205	0.00000	0.00000
	0.00000	0.00000	0.00000	0.00000	0.00000	0.00000	0.00000	0.00202	0.00000	0.00000
	0.00000	0.00000	0.00000	0.00000	0.00000	0.00000	0.00000	0.00000	0.00000	0.00000
	0.00000	0.00000	0.00000	0.00000	0.00000	0.00000	0.00000	0.00000	0.00000	0.00000
	0.00000	0.00000	0.00000	0.00000	0.00000	0.00000	0.00000	0.00000	0.00000	0.00000

(B)

Week 20 % human CD45+ reconstitution in BM of 1° recipient								
CD34+ cell dose	2000		500		200		50	
Group	Ctrl	JNK-IN-8	Ctrl	JNK-IN-8	Ctrl	JNK-IN-8	Ctrl	JNK-IN-8
% human CD45+	70.34632	69.05340	4.27980	32.52033	23.33849	0.76121	0.08969	16.60027
	43.48534	58.12325	3.74332	1.84134	5.40044	0.70683	0.08326	8.43525
	23.78168	46.65842	0.71267	0.47325	3.20442	0.61670	0.07635	0.17407
	9.29757	6.05346	0.17668	0.25963	0.22422	0.53243	0.07529	0.16791
	1.77170	1.93262	0.09148	0.25529	0.12511	0.53135	0.06700	0.14749
	1.17307	1.10487	0.08797	0.23703	0.10237	0.46492	0.06383	0.11497
	1.14679	0.76336	0.08643	0.19894	0.09524	0.45736	0.05962	0.11366
	0.73749	0.74610	0.08160	0.19828	0.07575	0.42644	0.05901	0.07182
	0.64891	0.39841	0.06275	0.18099	0.06568	0.36452	0.05608	0.06849
	0.51077	0.30558	0.05476	0.16361	0.06525	0.31378	0.05467	0.05162
	0.47659	0.28300	0.04809	0.15674	0.06476	0.27760		
	0.46245	0.15046	0.03364	0.13605	0.05419	0.23581		
	0.21028	0.00860	0.02840	0.13041	0.05111	0.20463		
	0.08272		0.01768	0.12670	0.02847	0.18287		
	0.05190				0.00857	0.15721		

(C)

Week 14 % human CD45+ reconstitution in PB of 2° recipient						
Cell dose (from 1° recipient BM)	10000000		1000000		100000	
Group	Ctrl	JNK-IN-8	Ctrl	JNK-IN-8	Ctrl	JNK-IN-8
% human CD45+	0.02400	0.21000	0.03000	0.04000	0.01100	0.01700
	0.00137	0.18000	0.01300	0.19000	0.02000	0.02700
	0.05200	0.15000	0.04000	0.06200	0.01300	0.01600
	0.02000	0.12000	0.00987	0.11000	0.00989	0.00979
	0.03300	0.11000	0.08600	0.21000	0.01400	0.00397

Supplemental Table 3. List for qPCR primers.

Gene	Accession. No	Primer Sequence
β-actin	NM_001101	Forward, 5’-CATGTACGTTGCTATCCAGGC-3’
		Reverse, 5’-CTCCTTAATGTCACGCACGAT-3’
CCNG2	NM_004354	Forward, 5’-TCTGTATTAGCCTTGTGCCTTCT-3’
		Reverse, 5’-CCTTGAAACGATCCAAACCAAC-3’
CDKN1B	NM_004064	Forward, 5’-TAATTGGGGCTCCGGCTAACT-3’
		Reverse, 5’-TGCAGGTCGCTTCCTTATTCC-3’
CDKN1C	NM_001122630	Forward, 5’-GCGGCGATCAAGAAGCTGT-3’
		Reverse, 5’-GCTTGGCGAAGAAATCGGAGA-3’
HIF1A	NM_181054	Forward, 5’-GAACGTCGAAAAGAAAAGTCTCG-3’
		Reverse, 5’-CCTTATCAAGATGCGAACTCACA-3’
ATM	NM_000051	Forward, 5’-GGCTATTCAGTGTGCGAGACA-3’
		Reverse, 5’-TGGCTCCTTTCGGATGATGGA-3’
PDK2	NM_001199899	Forward, 5’-ATGAAAGAGATCAACCTGCTTCC-3’
		Reverse, 5’-GGCTCTGGACATACCAGCTC-3’
FOXO3	NM_001455	Forward, 5’-TCACGCACCAATTCTAACGC-3’
		Reverse, 5’-CACGGCTTGCTTACTGAAGG-3’
cJun	NM_002228	Forward, 5’-GTGACGGACTGTTCTATGACT-3’
		Reverse, 5’-GGGTTACTGTAGCCATAAGGT-3’

Supplemental Figure 1

A

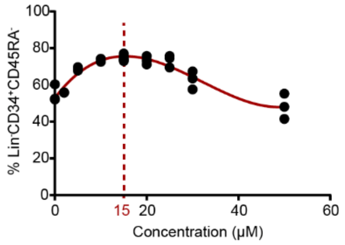

C

Group	Cell dose	Tested	Engrafted	SRC frequency	p value
**DMSO**	5,000	4	4	1/1796	(vs. uncultured) 0.918
	1,000	5	2		
	200	5	0		
**Uncultured**	5,000	5	4	1/1682	
	1,000	4	4		
	200	5	0		
**JNK-IN-8**	5,000	5	5	1/125	(vs. uncultured) 0.000609<br>(vs. DMSO) 0.000794
	1,000	1	1		
	200	5	4		

B

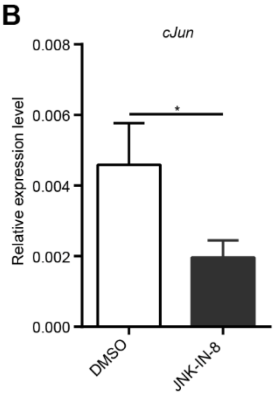

D

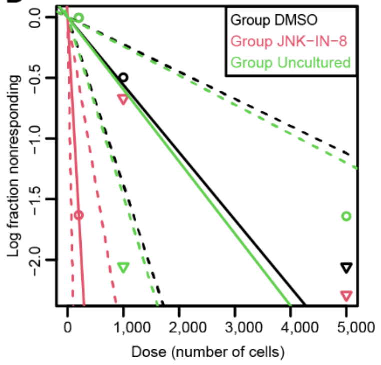

Supplemental Figure 2

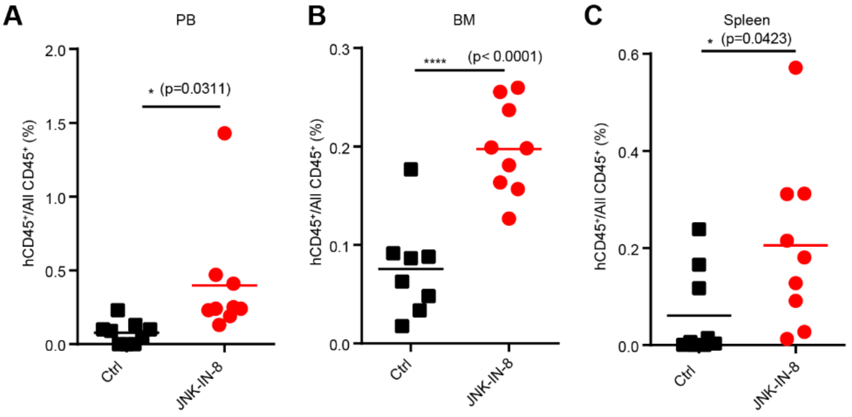

D

Group	Cell dose	Tested	Engrafted	1°SRC frequency	p value
Ctrl	2,000	15	13	1/934	6.64E-10
	500	14	4		
	200	15	6		
	50	10	0		
JNK-IN-8	2,000	13	12	1/148	
	500	14	14		
	200	15	15		
	50	10	7		

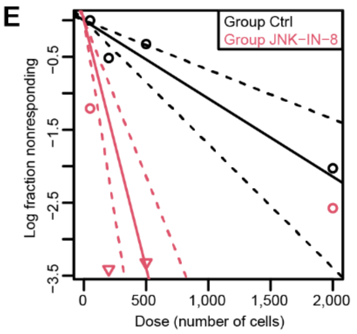

F

Group	Cell dose	Tested	Engrafted	2°SRC frequency	p value
Ctrl	10,000,000	5	4	1/1626087	1.34E-04
	1,000,000	5	4		
	100,000	5	4		
JNK-IN-8	10,000,000	5	5	1/108951	
	1,000,000	5	5		
	100,000	5	3		

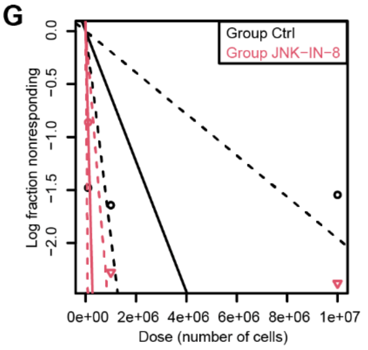

Supplemental Figure 3

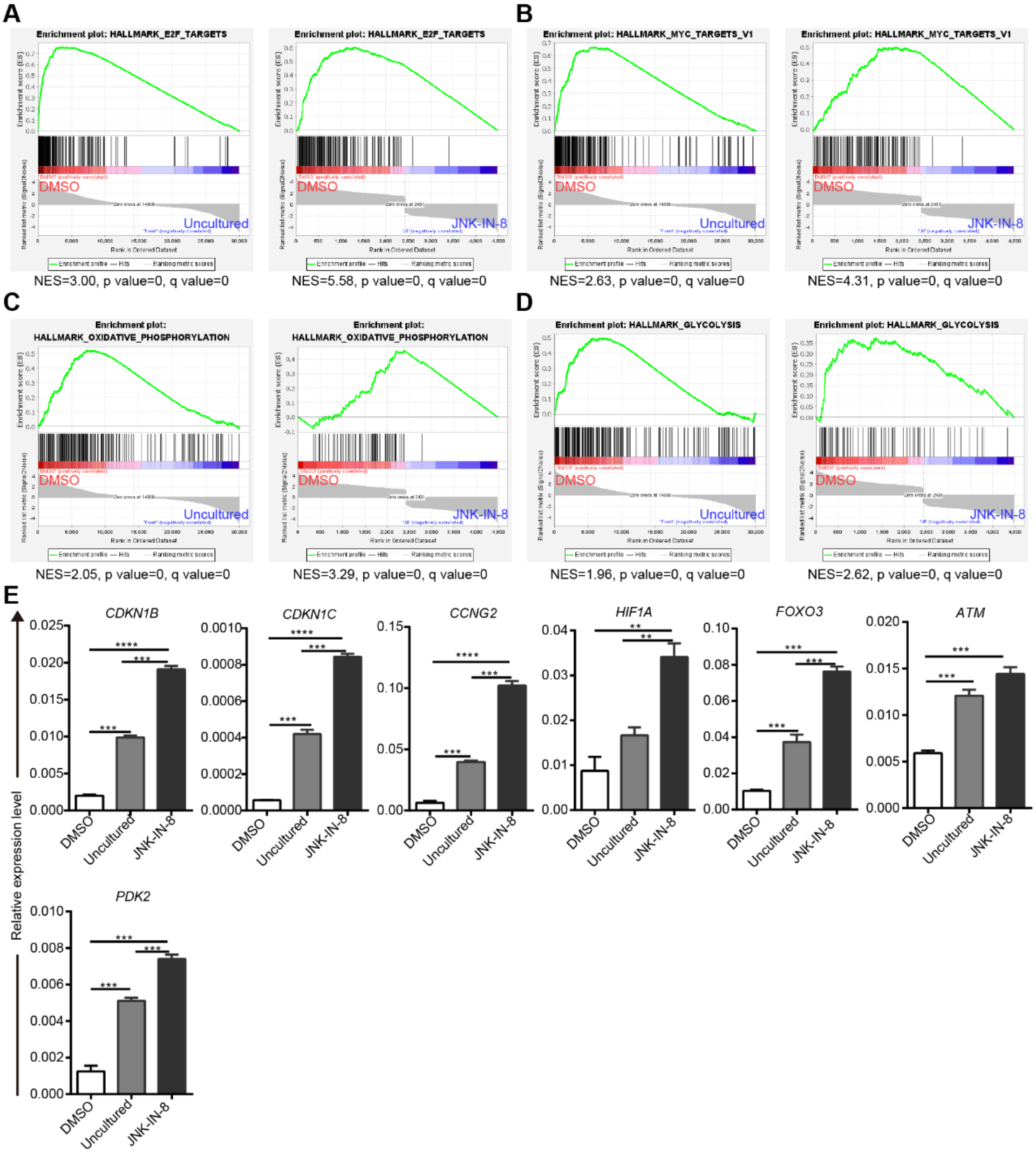

1
